# A Study of Electrochemical Machining of Ti-6Al-4V in NaNO_3_ solution

**DOI:** 10.1038/srep35013

**Published:** 2016-10-13

**Authors:** Hansong Li, Chuanping Gao, Guoqian Wang, Ningsong Qu, Di Zhu

**Affiliations:** 1 College of Mechanical and Electrical Engineering, Nanjing University of Aeronautics and Astronautics, Nanjing 210016, China

## Abstract

The titanium alloy Ti-6Al-4V is used in many industries including aviation, automobile manufacturing, and medical equipment, because of its low density, extraordinary corrosion resistance and high specific strength. Electrochemical machining (ECM) is a non-traditional machining method that allows applications to all kinds of metallic materials in regardless of their mechanical properties. It is widely applied to the machining of Ti-6Al-4V components, which usually takes place in a multicomponent electrolyte solution. In this study, a 10% NaNO_3_ solution was used to make multiple holes in Ti-6Al-4V sheets by through-mask electrochemical machining (TMECM). The polarization curve and current efficiency curve of this alloy were measured to understand the electrical properties of Ti-6Al-4V in a 10% NaNO_3_ solution. The measurements show that in a 10% NaNO_3_ solution, when the current density was above 6.56 A·cm^−2^, the current efficiency exceeded 100%. According to polarization curve and current efficiency curve, an orthogonal TMECM experiment was conducted on Ti-6Al-4V. The experimental results suggest that with appropriate process parameters, high-quality holes can be obtained in a 10% NaNO_3_ solution. Using the optimized process parameters, an array of micro-holes with an aperture of 2.52 mm to 2.57 mm and maximum roundness of 9 μm were produced using TMECM.

Titanium alloys have multiple applications in the automobile industry, mechanical machining, medical equipment, and other industries. This diversity of functionality is because of titanium’s low density, high strength, and corrosion resistance^1^,^2^. However, these properties also make it difficult to machine Titanium Alloy Ti-6Al-4V into complex-shaped components. Thus many studies have been undertaken to explore how to machine titanium alloys like Ti-6Al-4V into desired shapes. C. L. Qiu, G. A. Ravi, C. Dance *et al*. have used direct laser disposition to create Ti-6Al-4V aerostructures that are high in quality and performance, and tend to experience significantly reduced levels of strain^3^. M. D. Moses and M. P. Jahan made blind holes, micro-through-holes, and grooves in Ti-6Al-4V using micro electrical discharge machining (micro-EDM)^4^. Research by F. Z. Wang, J. Zhao, A. h. Li *et al*. looked at the processing characteristics of Ti-6Al-4V with high-speed side milling and showed that the work surface had high quality when the cutting speed was 200 m/min and a small cutting force was used^5^.

Electrochemical machining (ECM) is a non-traditional machining method which allows applications to all kinds of metallic materials regardless of their mechanical properties. It can be used to machine metals into complex-shaped components no matter how hard the metals are^6^. An ECM process is usually free of machining stress and cathode wear, and is capable of offering good surface finish^7^,^8^. ECM is widely applied to the machining of Ti-6Al-4V components, because of these advantages. A 20 μm × 20 μm array of pits was machined into titanium by C. Madore, O. Piotrowski, and D. Landol using TMECM^9^. Micro-holes with an aperture of 100 μm were made in titanium through a TMECM process by P. Kern, J. Veh1, and J. Michler^10^.

Electrolyte properties affect the dissolution of the materials being machined^11^. The electrolytes in an ECM system not only transfer currents during machining, but also carry away the by-products and heat that are produced in the machining process. The electrolytes used in ECM can greatly influence the processing quality. Different electrolytes can produce different machining effects when applied to the same material and a particular electrolyte’s machining effect varies with the type of material that is machined.

A great deal of research has examined the processing characteristics of Ti-6Al-4V in different electrolytes. One study by M. Weinmann, M. Stolpe *et al*. found that the ECM process can be accelerated by increasing the concentration of chloride ions in the electrolytes^12^. N. S. Qu, X. L. Fang *et al*. have applied a multicomponent electrolyte containing 10% NaNO_3_ and 10% NaCl to ECM of Ti-6Al-4V by pulsating electrolytes; the minimum roughness of the resulting surfaces (12 mm × 12 mm) was Ra 0.53 μm^8^. P. Sudhakar Rao, P. K. Jain, and D. K. Dwivedi^13^ investigated the influence of process parameters in electro chemical honing on the finish of the hole surfaces in Ti-6Al-4V using a mixture of the electrolytes NaCl and NaNO_3_. An excellent surface finish was achieved with a multicomponent electrolyte containing 3 parts NaCl to 1 part NaNO_3_. Through jet micro-electrochemical machining in an NaBr electrolyte, X. Lu and Y. Leng^14^ produced micro holes in a cylindrical work-piece, whose apertures were several hundred micrometers and aspect ratios were higher than 1.3. In a study by M. Schneider, S. Schroth, S. Richter *et al*.^15^ mirror-finish surfaces were produced by ECM of titanium in a eutectic mixture of choline chloride and ethylene glycol; they achieved a current efficiency of approximately 80%.

The studies detailed above have yielded superior results. However, it is difficult to renew and maintain multicomponent electrolytes. This can be avoided by using single-electrolyte solution because single-electrolyte solution can be reused after impurities are filtered out and electrolytic cells are replenished. For this reason, this study used a 10% NaNO_3_ solution as a single-electrolyte solution to create multiple holes in Ti-6Al-4V sheets by TMECM. The polarization curve and current efficiency curve of Ti-6Al-4V in a 10% NaNO_3_ solution were measured in order to discover its electrical properties in this electrolyte.

Then, an orthogonal TMECM experiment was conducted on Ti-6Al-4V in a 10% NaNO_3_ solution, based on the measurements and the fact that the non-processing region in TMECM is covered and protected from corrosion. The effects of electric parameters plus the interactions between them on the processing results were investigated. Diameter uniformity and surface finish were used as the evaluation standards for analysis. Subsequently, optimized parameters were determined and a micro-holes array was fabricated on the thin Ti-6Al-4V sheet.

## Measurements of the Processing Characteristics of Ti-6Al-4V

Polarization voltage refers to the additional voltage required to dissolve a metallic material^11^. Measuring the polarization curve of Ti-6Al-4V in a 10% NaNO_3_ solution can help to determine the range of processing voltage needed for the experiment. If the processing voltage reaches the polarization voltage during ECM, then the dissolution rate of the material depends on the current density in the electrolyte^11^. Since the current efficiency curve of the alloy in this electrolyte solution can provide information about the dissolution rate of the material at different current densities, it is possible to determine the proper processing voltage on the basis of the values of the current density on this curve. Therefore, it is necessary to measure the polarization curve and current efficiency curve of Ti-6Al-4V in a 10% NaNO_3_ solution.

### Measurement of polarization curve

**Figure 1:**
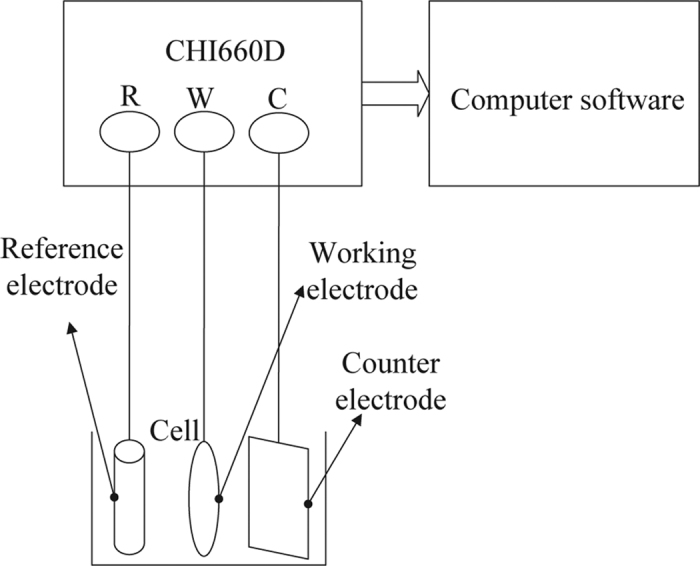
Schematic of the experimental equipment.

**Figure 2:**
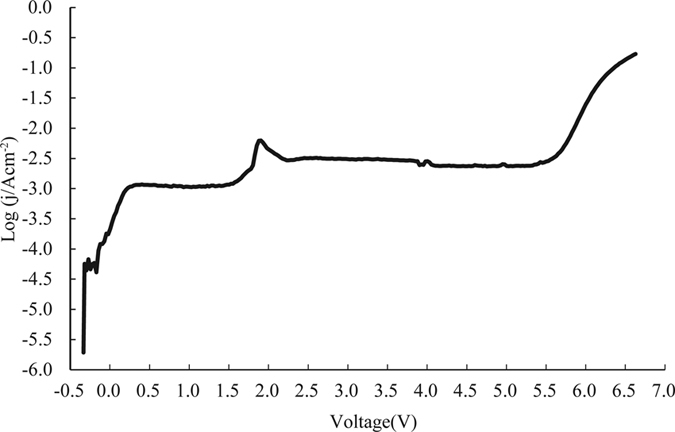
Polarization curve of Ti-6Al-4V in a 10% NaNO_3_ electrolyte solution.

Corrosion current density is commonly used as an index to measure how active a corrosion reaction is. Corrosion rate is normally in direct proportion to the corrosion current density on the polarization curve^16^. In this experiment the polarization curve of Ti-6Al-4V was measured in order to determine the proper processing voltage for this material. On each point of the polarization curve the abscissa is the anode potential, denoted as E, and the ordinate is the anode current density, denoted as I. This curve reflects the dissolution behavior of the alloy, i.e. the active region and passive region^17^. The experiment was conducted in a CHI660D electrochemical workstation and a 10% NaNO_3_ solution was used as the single-electrolyte solution. A square Ti-6Al-4V sheet with dimensions of 10 mm × 10 mm × 0.5 mm was used as the work-piece electrode, a platinum net was used as the counter electrode, and a calomel electrode was used as the reference electrode. During measurement, the voltage increased from −3 V to 7 V at a scanning rate of 1 mv/s. Figure 1 shows the schematic of the experimental equipment and Fig. 2 illustrates the experimental results.

As the polarization curve (Fig. 2) shows, there is almost no increase in the anode current in the voltage range of −3 to 5.24 V. This is primarily because the oxide film on the surface of the work-piece prevented the anode from dissolving^1^. The corresponding region is referred to as a passive region. As the voltage increases from 5.24 to 6.6 V, the current shows a sharp and continuous increase until it reaches a maximum value, indicating that the oxide layer on the surface of the work-piece has been removed^18^,^19^. The corresponding region is considered to be a transpassive region; it should be followed by a stable region of dissolution. It is reasonable to infer from the polarization curve that the breakdown voltage of the work-piece was 5.24 V and the voltage required to initiate the machining of Ti-6Al-4V (the initial voltage) was 6.6 V.

### Measurement of current efficiency curve

**Figure 3:**
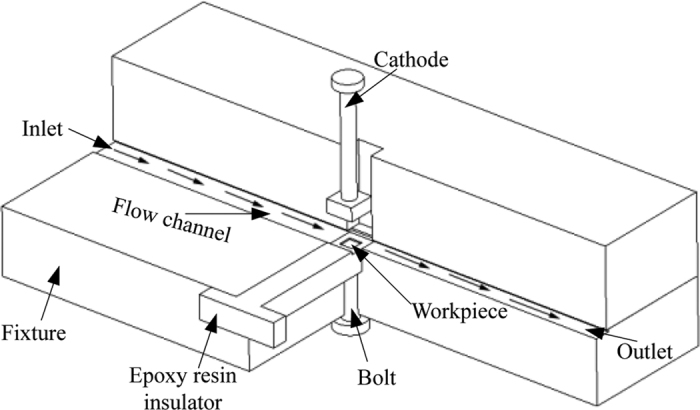
Schematic of the current-efficiency measuring equipment.

Current density and current efficiency affect the dissolution rate of metal material together. For a certain kind of material, current efficiency varies with the current density, and subsequently affects the quality of surface^20^. For TMECM, the current densities on the material surface are different. Accordingly, it is essential to measure the dependence of current efficiency on the current density. Further, the current efficiency curve is very important in actual machining processes. It is therefore necessary to measure the current efficiency curve of Ti-6Al-4V in a 10% NaNO_3_ solution. To achieve this, a piece of equipment was designed and fabricated for use in this experiment– its schematic is shown in Fig. 3. A square sheet work-piece with the dimensions 10 mm × 10 mm × 0.5 mm was placed in an insulating tank made from an epoxy resin. A bolt was used as both the work-piece electrode to connect the work-piece to the anode and as a fastener to fix the work-piece to the insulating tank. The tank had a square opening measuring 8 mm × 8 mm, which was the region to be machined. The rest of the tank was carefully designed to be leak proof, ensuring the accuracy of measurement. The gap between the cathode and work-piece could be adjusted by moving the cathode up and down. In this experiment the gap was set at 1 mm. Electrolyte could flow through the flow channel to carry away the by-products of machining. In this experiment, the constant current method was applied to measure current efficiency, which is given by:


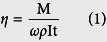


where, M is the mass of material removed from the work-piece (g); I is the current (A); t is the processing time (s); *ω* is the theoretical value of the volume electrochemical equivalent (cm^3^A^−1^S^−1^); and *ρ* is the material density (g/cm^3^).

**Figure 4:**
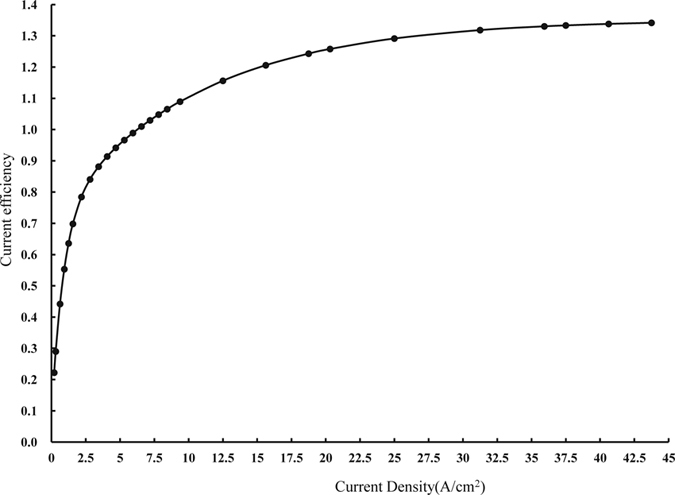
Current efficiency curve of Ti-6Al-4V in 10% NaNO_3_ electrolyte.

In each experimental run, the current was increased gradually. Each current value was used for a fixed processing time and the measurement was repeated three times in order to reduce error. The work-piece was cleaned, dried, weighed, and sealed before and after each experimental run so that the mass loss of the work-piece, M, could be obtained. The measurements were then plotted as a current efficiency curve (Fig. 4).

During the experiment, the current density fell within the range of 0.2 to 43.75 A·cm^−2^ and the processing time was meticulously controlled and recorded. Figure 4 illustrates the current efficiency curve of Ti-6Al-4V in a 10% NaNO_3_ solution. It can be inferred from the figure that current density did not exert significant influence on this alloy. The current efficiency of Ti-6Al-4V reached a high of 0.64 at a low current density of 1.25 A·cm^−2^ and then it reached 1.0 when the current density increased to 6.56 A·cm^−2^. As the current density further increased to 43.75 A·cm^−2^, the alloy’s current efficiency increased up to 1.34, which was the maximum level recorded in this experiment. Because the components of Ti-6Al-4V differ in activity level and thus differ in dissolution rate, the processing region underwent non-uniform dissolution, leading to mechanical spalling of the material and resulted in an uneven work surface^21^. This finding is consistent with the results of a study by C. Y. Yu, Y. S. Yang, C. K. Cheng *et al*.^21^, in which Ti-6Al-4V was machined in a 20% NaNO_3_ electrolyte solution. As the mechanical spalling was not caused by the ECM, the reduction in the mass of the work-piece should be larger than the actual mass of material removed by the ECM. Therefore, the current efficiency could exceed 1.0. However, when the current density was below 0.128 A·cm^−2^, the work-piece did not dissolve and the corresponding current efficiency was 0.

According to the obtained polarization curve, the voltage reached the initial voltage for the ECM of Ti-6Al-4V, at 6.6 V, when the current was 0.294 A. At this point the current density was 0.294 A·cm^−2^ and the corresponding current efficiency was 0.29. As the processing voltage increased beyond 6.6 V, the current efficiency of Ti-6Al-4V gradually increased, indicating that this alloy is machinable in this electrolyte solution.

## TMECM Experiment

### The principle of TMECM

**Figure 5:**
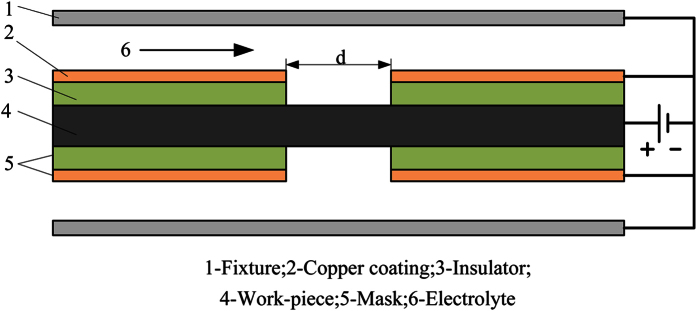
Schematic of through-mask electrochemical machining.

**Figure 6:**
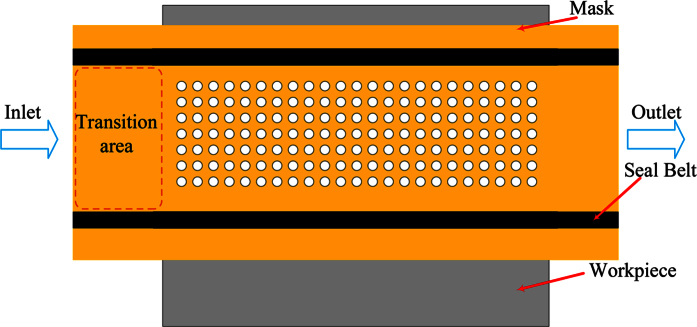
The image of machined surface.

Through-mask electrochemical machining (TMECM) is an electrochemical machining method in which a work-piece is placed between two horizontal masks shaped in the desired form. Figure 5 illustrates the principle of TMECM, and the image of machined surface is shown in Fig. 6. A rectangular inlet was designed for electrolyte solution flowing into processing area. The long enough transition area was designed to make the flow field uniform. A mask consists of a piece of insulator and the copper coating on its surface. The electrolyte can flow through the gaps between the fixtures and copper coatings. The regions on the work-piece that are exposed by the openings in the masks are subject to corrosion, while the regions covered by the masks are protected from corrosion. During the TMECM process the work-piece is tightly held by the upper and lower masks. Both the fixtures and masks are connected to the negative pole of a current source and the work-piece is connected to the positive pole. As both the upper and lower surfaces of the work-piece are machined simultaneously, the micro-holes that are produced have small tapers and steep walls.

**Figure 7:**
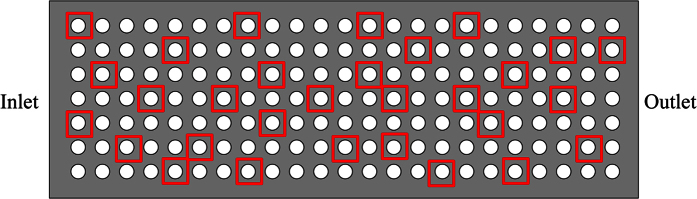
Image of the holes selected for measuring.

According to the polarization curve, as processing voltage is above 6.6 V, samples of Ti-6Al-4V dissolve in a 10% NaNO_3_ electrolyte solution. Furthermore, the current efficiency is approximate 0.3 when dissolution is initiated based on the current efficiency curve. Therefore, an orthogonal array (Table 1) was designed for this experiment to study the effects of voltage, pulse frequency, and duty ratio on processing quality in TMECM of Ti-6Al-4V. Upon the completion of the experiment, an ultrasonic cleaner was used to clean the work-piece. Later on, as is shown in Fig. 7, thirty holes marked by red square were selected randomly from the machined hole-array for measuring. Then, the work-piece was observed under a Leica microscope to examine the surface quality of the resulting micro-holes and to measure their diameters. During measurement, some points were picked from circumference of each hole to measure the diameter.

Then the tapers of the micro-holes were measured with an OMS400, which is a type of trilinear coordinate measuring instrument produced by Mahr, a German company.

### Experimental process

This orthogonal experiment used a 10% NaNO_3_ solution as a single-electrolyte solution. The electrolyte was filtered throughout the machining process in order to ensure its purity and avoid the influence of any impurity on the processing quality. Throughout the whole process the electrolyte pressure was maintained at 0.6 MPA and the temperature were maintained at 45 °C, respectively, in the whole process. A chronograph was used to record processing time; all experimental runs were equal in duration. The current was recorded at 10 second intervals during the process.

In Table 1, the voltage, pulse frequency, and duty ratio are represented by A, B, and C, respectively. AB represents the interaction between voltage and pulse frequency, AC denotes the interaction between voltage and duty ratio, and BC represents the interaction between pulse frequency and duty ratio. In column A, 15 V, 25 V, and 35 V are voltage level 1, level 2, and level 3, respectively. In column B, 200 HZ, 400 HZ, and 600 HZ denote pulse frequency level 1, level 2, and level 3, respectively. In column C, 20%, 30%, and 40% represent duty ration level 1, level 2, and level 3, respectively.

Our experiment used diameter uniformity of the produced micro-holes as an index to evaluate processing quality. The diameter uniformity can be calculated using the following formula:


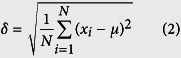


where, N is the total number of micro-holes, *x* is the micro-hole diameter (mm), and *μ* is the average diameter of all micro-holes.

In the analysis of the experimental results, the processing quality was first evaluated in terms of the diameter uniformity of the micro-holes. The three combinations of levels that exhibited the highest diameter uniformity were selected from all of the combinations listed in the orthogonal array, and then the optimal process parameters of the three combinations were determined. Later, the processing quality was assessed using the three sets of parameters in terms of surface finish of the micro-holes, and one set of parameters was chosen from the optimal process parameters of the three combinations. The process parameters obtained through the two steps were the process parameters that were optimized in this experiment.

## Results and Discussions

An orthogonal experiment was performed with the experimental design presented in Table 1. The results of the processing experiment are summarized in Table 2. After the measurement of the diameter with Leica, no obvious regular nonuniformity was observed. On one hand, that was due to the long enough transition area and the high electrolyte pressure. On the other hand, the number of rows of the hole-array was small and it was fixed in the middle of the processing area. As shown in the table, the three parameters and the interactions between them were ranked in descending order of range, R, as follows: A > A × B > B × C > A × C > B > C. This relationship suggests that voltage was the factor that had the greatest effect on diameter uniformity, followed by the interaction between voltage and pulse frequency. Duty ratio was the least influential factor.

In orthogonal experiment, Ki is the sum of experimental results of a particular factor at level i. In this paper, experimental result is diameter uniformity. Column A in the table above shows that K1, K2, and K3 decreased as the voltage A increased, indicating that a higher voltage contributed to a higher diameter uniformity, or the diameter uniformity was higher at a voltage of 35 V than at the other two voltage levels. Column B shows that the value of Ki decreased and then increased as the pulse frequency increased. This suggests that a moderate increase in pulse frequency can improve the diameter uniformity, but if the pulse frequency increased beyond a certain value, the uniformity tended to decrease. The reasons for the results may be as follows.

As the voltage increased, the electric field intensity increased and the electric field distribution became more uniform. At high voltages, the amounts of material removed from the micro-holes over the same period of time were roughly equal, indicating that the corrosion rates at the holes were similar. Therefore, the micro-hole diameters had little variation and high diameter uniformity was achieved.

When the pulse frequency of the current source was relatively low, the intervals between electrical discharges were long enough for the electrolyte to carry away the heat and processing by-product. As a result, the electrolyte in the processing region had uniform electric conductivity and a stable temperature, thus ensuring an efficient and continuous machining process. This further contributed to the uniform diameters of the resulting micro-holes. As the pulse frequency increased, the intervals between electrical discharges shortened. When the intervals were too short for the electrolyte to remove the resulting heat and by-product efficiently, the temperature of the processing region increased and the electrolyte’s electric conductivity varied significantly, resulting in greater variations in hole diameter.

At low duty ratios, the heat and by-products generated in machining were completely discharged together with the electrolyte, ensuring a stable machining process. As the duty ratio increased to the level that inhibited the discharge of heat and product, the temperature of the processing region gradually rose and the electrolyte around the holes could not be replaced by adequate fresh electrolyte. This worsened the machining condition and reduced the uniformity of the distribution of electric field and flow field. Consequently, the stable machining process was disrupted and the diameters of the micro-holes became non-uniform.

In order to select optimal combinations, the result of each combination was the sum of the experimental results of the factors at corresponding levels. Tables 3 and 4 show the result of the combinations of A × B and B × C, respectively.

**Figure 8:**
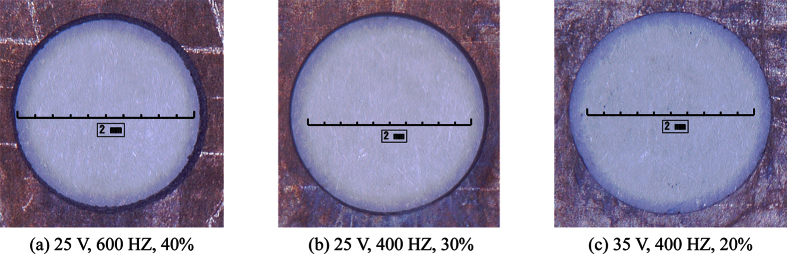
Morphology of micro-holes obtained using the three selected sets of parameters.

**Figure 9:**
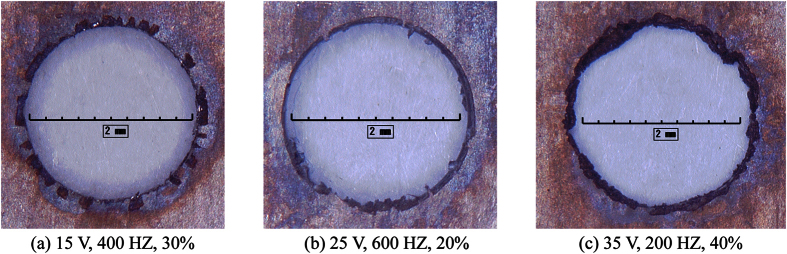
Morphology of micro-holes obtained using other process parameters.

Higher diameter uniformity is associated with smaller standard deviations. Tables 3 and 4 show that among all combinations, the combination of A3B2 and B2C1 has the smallest values, which are 0.0222 and 0.0251, respectively; this corresponds to the parameter combination of 35 V × 400 HZ and 400 HZ × 20%. The combination of A2B3 and B3C3 has the second smallest values, of 0.0235 and 0.0300, respectively; the corresponding parameter combination is 25 V × 600 HZ and 600 HZ × 40%. The next smallest is the combination of A2B2 and B2C2 at 25 V × 400 HZ and 400 HZ × 30%, with values of 0.0260 and 0.0322, respectively. The optimal process parameters of the three combinations were determined and are summarized in Table 5. Later, the micro-holes machined using the three sets of process parameters were assessed in terms of surface finish and were then compared. Figure 8 shows the surface morphology of the micro-holes produced using the three sets of parameters, and Fig. 9 shows the surface morphology of some of the micro-holes obtained using other process parameters.

As Fig. 8 shows, the micro-holes that were machined using the previously discussed three sets of optimal process parameters featured high-quality surface finish and there was no obvious evidence of corrosion on their surfaces. However, the micro-holes obtained using the combination of A2B3 and B3C3 (Fig. 8(a)) had a much larger taper than the tapers of the micro-holes obtained using the other two combinations. The reasons were as follows. The middle processing voltage (25 V) produced a certain current density corresponding to the current efficiency curve. The pulse-on time in a pulse period was the reciprocal of frequency multiplied by duty ratio. Activation needs a certain time to initiate^22^, which was called activation time. When the pulse-on time is close to the activation time, the current efficiency decreases greatly regardless of the voltage magnitude for just a small amount of time is used for metal dissolution. That means much more time is needed for workpiece to penetrate. For machining time is fixed, less remaining time is used for radial dissolution, which accounts for the high taper.

The taper of the micro-hole that was created with the combination of A2B2 and B2C2 (Fig. 8(b)) was larger than that of the hole made with the other combinations (Fig. 8(c)). The reason was similar to the one in Fig. 8a. The difference was that the product of the reciprocal of pulse frequency multiplied by duty ratio was higher, leading to a lower taper relative to Fig. 8a.

The micro-hole in Fig. 8(c) has a good surface finish, and its taper is essentially invisible to the naked eye. The activation time decreases in a certain range as the processing voltage increases^22^. The high processing voltage created a uniformity electric field around the holes, which contributed to a good-shape circumference. On the other hand, the product of the reciprocal of pulse frequency multiplied by duty ratio was higher than the activation time at the processing voltage = 30 V, resulting in the remaining time was enough to minimize the taper.

The micro-holes machined using other process parameters (Fig. 9) exhibited poor surface stray current corrosion. When processing voltage was not high enough, the electric field around the hole was not uniform. That meant that the hole couldn’t be round enough and that the amount of the material dissolved around the circumference was different. In addition, activation time was longer at lower processing voltage. This caused non-circular serrated hole and bumpy surface (Fig. 9a), which was worse compared to Fig. 8b with a product of the reciprocal of pulse frequency multiplied by duty ratio equal to that in Fig. 9a. Compared with the parameters in Fig. 8a, the parameters in Fig. 9b were the same except the lower duty ratio. Therefore, the activation time was the same due to the same processing voltage while the product of the reciprocal of pulse frequency multiplied by duty ratio in Fig. 9b was lower. This caused a shorter metal dissolution time and consequently a lower current efficiency. Thus, the machining shape in Fig. 9b was worse than that in Fig. 8a and some protrusions appeared around the circumference. As for Fig. 9c, high processing voltage meant a lower activation time and the pulse-on time was longer than that in Fig. 8c. For the voltages in both Fig. 9c and Fig. 8c were the same, the activation times were the same. Therefore, the current efficiency in Fig. 9c was higher than Fig. 8c. Nevertheless, heat and by-products couldn’t be washed out in time, resulting in bad processing environment and non-uniform electric field. Therefore, non-circular holes were machined and the surface was not smooth.

Evaluations using two indices showed that the micro-holes obtained with the optimal process parameters of the combination of A3B2 and B2C1 (i.e. 35 V, 400 HZ, and 20%), exhibited perfect surface morphology and no noticeable stray current corrosion, and the micro-hole’s taper was too small to be visible to the naked eye.

### Optimization of process parameters

**Figure 10:**
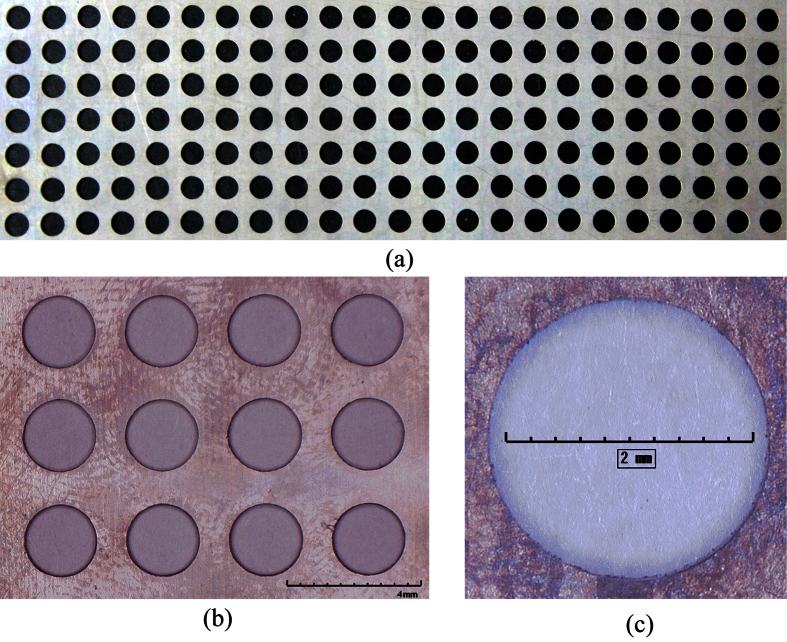
Images of machined hole-array: (**a**) Photograph of the array holes; (**b**) Local amplification microscopy image of the array holes; (**c**) microscopy image of a single hole.

Using appropriate process parameters can improve both machining precision and efficiency. An analysis of the results of the orthogonal experiment suggests that a TMECM process using a 10% NaNO_3_ solution is capable of producing micro-holes with uniform diameters, high surface quality, good morphology, and small tapers, when the voltage is 35 V, pulse frequency is 400 HZ, and duty ratio is 20%. Using the optimized process parameters, an array of micro holes (Fig. 10) was machined into a Ti-6Al-4V sheet by TMECM in a 10% NaNO_3_ solution. Figure 10a was the image of the array holes, and Fig. 10b was local amplification microscopy. Microscopy image of a single hole was shown in Fig. 10c. The figure below shows that the resulting micro-holes were characterized by high-quality surface finish and good morphology. The diameters of all these holes in the array were examined using a Leica microscope, and their average diameter was calculated as Φ 2.54 mm with the deviation was 0.05 mm. The maximum roundness of these holes was 9 μm when measured with an OMS400 trilinear coordinates measuring instrument.

## Conclusions

In order to solve the problem that it is inconvenient to renew the multicomponent electrolyte solution for ECM of TC4, a 10% single- electrolyte NaNO_3_ solution was used to fabricate micro holes by TMECM. An analysis of the experimental data can lead to the following conclusions:

1. The electric electrochemical characteristics of TC4 in NaNO_3_ solution were researched and polarization curve and current efficiency curve were obtained.
2. A TMECM orthogonal experiment was conducted on Ti-6Al-4V sheets in a 10% NaNO_3_ solution, in order to study the influences of electric parameters as well as their mutual interactions upon the processing quality. Micro-hole diameter variation and processing quality were used in analysis of the experimental results. Based on the evaluation, the optimal process parameters were determined with which a micro-holes array was machined on the TC4 sheet.
3. Ti-6Al-4V can be machined by TMECM in a 10% single-electrolyte NaNO_3_ solution. Stress should be put on the loss of NaNO_3_ solution and the elimination of by-products in the future research.

## Additional Information

**How to cite this article**: Li, H. *et al*. A Study of Electrochemical Machining of Ti-6Al-4V in NaNO_3_ solution. *Sci. Rep*. **6**, 35013; doi: 10.1038/srep35013 (2016).

## Figures and Tables

**Table 1:** Orthogonal array for the experiment.

Column number	Factor						
	1	2	3	4	5	6	7
Test number	A	B	AB		C	AB	BC
1	15	200	1		20	1	1
2	15	200	1		30	2	2
3	15	200	1		40	3	3
4	15	400	2		20	1	2
5	15	400	2		30	2	3
6	15	400	2		40	3	1
7	15	600	3		20	1	3
8	15	600	3		30	2	1
9	15	600	3		40	3	2
10	25	200	1		20	2	1
11	25	200	2		30	3	2
12	25	200	3		40	1	3
13	25	400	1		20	2	2
14	25	400	2		30	3	3
15	25	400	3		40	1	1
16	25	600	1		20	2	3
17	25	600	2		30	3	1
18	25	600	3		40	1	2
19	35	200	3		20	3	1
20	35	200	3		30	1	2
21	35	200	3		40	2	3
22	35	400	1		20	3	2
23	35	400	1		30	1	3
24	35	400	1		40	2	1
25	35	600	2		20	3	3
26	35	600	2		30	1	1
27	35	600	2		40	2	2

**Table 2:** Results of the orthogonal experiment.

Column number	Factor						Index
Test number	A	B	AB	C	AB	BC	Uniformity
1	15	200	1	20	1	1	0.0083
2	15	200	1	30	2	2	0.0066
3	15	200	1	40	3	3	0.0151
4	15	400	2	20	1	2	0.0113
5	15	400	2	30	2	3	0.0163
6	15	400	2	40	3	1	0.0229
7	15	600	3	20	1	3	0.0195
8	15	600	3	30	2	1	0.0203
9	15	600	3	40	3	2	0.0106
10	25	200	1	20	2	1	0.0115
11	25	200	2	30	3	2	0.0184
12	25	200	3	40	1	3	0.0195
13	25	400	1	20	2	2	0.0080
14	25	400	2	30	3	3	0.0079
15	25	400	3	40	1	1	0.0101
16	25	600	1	20	2	3	0.0082
17	25	600	2	30	3	1	0.0065
18	25	600	3	40	1	2	0.0088
19	35	200	3	20	3	1	0.0173
20	35	200	3	30	1	2	0.0094
21	35	200	3	40	2	3	0.0072
22	35	400	1	20	3	2	0.0059
23	35	400	1	30	1	3	0.0080
24	35	400	1	40	2	1	0.0084
25	35	600	2	20	3	3	0.0108
26	35	600	2	30	1	1	0.0098
27	35	600	2	40	2	2	0.0111
K1	0.1310	0.1133	0.0799	0.1007	0.1047	0.1153	
K2	0.0989	0.0988	0.1151	0.1032	0.0975	0.0900	
K3	0.0878	0.1055	0.1227	0.1138	0.1154	0.1123	
Range (R)	0.0432	0.0145	0.0427	0.0130	0.0179	0.0253	

**Table 3:** Combinations of voltage and pulse frequency (A × B).

Factor	B1	B2	B3
A1	0.0300	0.0506	0.0504
A2	0.0492	0.0260	0.0235
A3	0.0339	0.0222	0.0316

**Table 4:** Combinations of pulse frequency and duty ratios (B × C).

Factor	C1	C2	C3
B1	0.0372	0.0344	0.0418
B2	0.0251	0.0322	0.0415
B3	0.0381	0.0367	0.0300

**Table 5:** Optimal process parameters of the three combinations.

Combination	A3B2 and B2C1	A2B3 and B3C3	A2B2 and B2C2
Optimal process parameters	35 V, 400 HZ, 20%	25 V, 600 HZ, 40%	25 V, 400 HZ, 30%
